# Influence of HLA on clinical and analytical features of pediatric celiac disease

**DOI:** 10.1186/s12876-019-1014-0

**Published:** 2019-06-13

**Authors:** Eva Martínez-Ojinaga, Marta Fernández-Prieto, Manuel Molina, Isabel Polanco, Elena Urcelay, Concepción Núñez

**Affiliations:** 1 Servicio de Gastroenterología y Nutrición Pediátrica, Hospital Universitario La Paz, Madrid, Spain; 2 Laboratorio de investigación en Genética de enfermedades complejas, Instituto de Investigación Sanitaria del Hospital Clínico San Carlos (IdISSC), C/ Profesor Martín Lagos s/n 28040, Madrid, Spain

**Keywords:** HLA-DQ, Clinical symptoms, Serology, Atrophy, Diagnosis

## Abstract

**Background:**

Celiac disease (CD) is triggered by gluten and related prolamines in genetically susceptible individuals. We aimed to investigate the influence of HLA-DQ genotypes in clinical, serological and histological features related to CD.

**Methods:**

A retrospective observational study was performed including 463 Spanish patients with biopsy-proven CD. Clinical, serological, histological and HLA-DQ genetic data were collected from each participant. The presence of a family history of CD was also considered. Bivariate (chi-square tests or the Fisher’s exact test) and multivariate (logistic regression after adjusting for age and sex) analyses were performed to assess the association between clinical and laboratory parameters with HLA-DQ.

**Results:**

A predominance of females (62%), classical clinical presentation (86%) and positive anti-transglutaminase 2/endomysium antibodies (99%) was observed in our sample, with a mean age at onset of 2.6 ± 0.1 years. Five percent of our patients were first-degree relatives of subjects with CD, with HLA-DQ genetics showing increased homozygosity of HLA-DQ2.5 (*p* = 0.03) and HLA-DQ8 (*p* = 0.09). In the non-CD family history group, an association between delayed disease onset and HLA-DQ8 carriage was observed (*p* < 0.001), besides an influence of HLA-DQB1*02 gene dosage on clinical presentation and severity of histological damage (after adjusting for age and sex, *p* = 0.05 and *p* = 0.02, respectively) and a trend towards presence of specific antibodies (*p* = 0.09). These associations could not be evaluated properly in the group of patients with affected first-degree relatives due to the small sample size.

**Conclusions:**

HLA-DQ genotypic frequencies differ slightly between CD patients depending on their family history of CD. In patients lacking CD first-degree relatives, carriage of HLA-DQ2.5 with double dose of HLA-DQB1*02 seems to be associated with classical clinical presentation and more severe histological damage.

## Background

HLA-DQ2.5 and HLA-DQ8 heterodimeric receptors are considered necessary to develop celiac disease (CD), a systemic disorder characterized by the enteropathy triggered after gluten or related prolamines ingestion [1]. This confers HLA-DQ genotyping a very high negative predictive value for CD diagnosis, making this practice very useful to help clinicians to discard CD [2, 3].

HLA-DQ receptors are encoded by *HLA-DQA1* (α chain) and *HLA-DQB1* (β chain) genes. Specifically, HLA-DQA1*05 and HLA-DQB1*02 encode the HLA-DQ2.5 receptor and HLA-DQA1*03 and HLA-DQB1*03:02 encode HLA-DQ8. Around 90–96% of CD patients carry HLA-DQ2.5 and almost all the remainders carry HLA-DQ8 [4]. Both are involved in CD pathogenesis. They appear on the surface of antigen presenting cells and show high affinity by deamidated gluten-derived peptides, which bind and present to CD4 T cells located in the lamina propia, initiating the inflammatory cascade that is characteristic of CD [5]. As a consequence, specific antibodies, mainly anti-transglutaminase 2 (TG2) and anti-endomysium (EMA) antibodies, are produced, both directed against TG2, which is responsible for gluten deamidation and becomes the main autoantigen of CD. But the capital change involves the intestinal damage that usually includes villous atrophy and is accompanied by clinical manifestations in most cases.

The binding properties to gluten-derived peptides and the capacity to elicit an immunological response depends on the specific HLA-DQ molecules present in each individual, existing also a dosage effect [6–8]. HLA-DQ2.5 can bind the largest repertoire of immunodominant gluten peptides and presents the highest ability to form stable complexes. Thus, individuals carrying the HLA-DQ2.5 heterodimer show the highest risk to develop CD, especially when bearing two HLA-DQB1*02 alleles (double dose). The risk decreases in individuals with HLA-DQ8 or only the HLA-DQB1*02 allele (HLA-DQ2.2 receptor), and is lowest in presence of only HLA-DQA1*05 (HLA-DQ7.5 receptor). Considering these differences, we hypothesized that HLA-DQ receptors could have a role on the clinical outcome and/or the serological and histological alterations present in each patient. However, little information exists regarding this issue, with some papers relating HLA genetics with some clinical and analytical characteristics but not others [9–14].

In a previous work, we studied the different CD risk conferred by HLA-DQ genotypes [15]. At this time, we have performed a retrospective observational study to analyze the possible influence of HLA-DQ genotypes on the clinical, analytical and histological manifestations at the onset of CD.

## Methods

### Study subjects

A total of 463 subjects with CD were included, all studied in a previous work [15]. These patients were diagnosed at the Gastroenterology Department of the Hospital La Paz between 1977 and 2011 according to the corresponding valid ESPGHAN criteria [1, 16]. All were children (0–14 years old) with Spanish ancestry.

### HLA-DQ genotyping and distribution

HLA-DQ genotyping was performed using PCR-SSOP (Polymerase Chain Reaction-Sequence Specific Oligonucleotide Probe) for *HLA-DRB1*, -*DQA1* and -*DQB1.* HLA-DQ data of our CD patients are shown in Table 1.

**Table 1:** HLA-DQ distribution in our celiac disease patients

HLA-DQ genotype	N	%
HLA-DQ2.5/DQ2.5	54	11.7
HLA-DQ2.5/DQ2.2	135	29.2
HLA-DQ2.5/DQ7.5	38	8.2
HLA-DQ2.5/DQX	138	29.8
HLA-DQ2.5/DQ8	20	4.3
HLA-DQ2.2/DQ7.5^a^	60	13.0
HLA-DQ8/DQ8	2	0.4
HLA-DQ8/DQ2.2, DQ7.5 or DQX	8	1.7
HLA-DQ2.2/DQX	7	1.5
HLA-DQ7.5/DQX	1	0.2

^a^HLA-DQ2.5 trans; HLA-DQX indicates an haplotype different from HLA-DQ2.5, HLA-DQ8, HLA-DQ2.2 and HLA-DQ7.5

### Clinical and analytical data

The following variables were extracted after reviewing inpatient and outpatient medical records: 1) sex; 2) age at onset, considered as the first time of reporting the clinical symptoms explained by CD; 3) clinical manifestations, being classified as classical symptoms (weight loss, growth retardation, chronic diarrhea, vomiting, hyporexia and abdominal distension) and non-classical symptoms (iron deficiency anemia, oral thrush, dermatitis herpetiformis or subclinical disease in individuals from risk groups such as first-degree relatives with CD or presence of associated conditions); 4) serological data: positive/negative IgA or IgG (in IgA deficient patients) anti-TG2 and/or EMA, and in case of positive anti-TG2, we also considered the antibody level by establishing two groups (≤ or > 10 times the upper limit of normality); 5) histology, graded following the Marsh-Oberhuber classification [17], with biopsies obtained before using this classification adapted as follows: normal histology as Marsh 0, lymphocytic enteritis as Marsh 1, mild partial atrophy as Marsh 2, moderate partial atrophy as Marsh 3a, intense partial atrophy as Marsh 3b and subtotal atrophy as Marsh 3c.

### Statistical analysis

The association between clinical and laboratory parameters, and between each of them and HLA was assessed by chi-square tests or the Fisher’s exact test, when appropriate, using 2 × 2 contingency tables (bivariate analysis) or by logistic regression after adjusting for age and sex (multivariate analysis). Comparisons related to age at onset were performed by means of the Mann-Whitney U test or the Kruskal-Wallis test depending on two or more groups were considered, respectively. Two-sided tests were always considered and significant associations were established at *p* values below 0.05. Analyses were performed with Statcalc (EpiInfo v6) or the statistical package SPSS v24.0.

## Results

Age at onset ranged from 7 months to 14 years in our 463 CD patients, with a mean of 2.6 ± 0.1 years. A predominance of females (62%) and classical clinical presentation (86%) was observed. All but two patients showed positive anti-TG2/EMA serology. Since CD was confirmed in all patients and anti-TG2 and EMA antibodies identify the same antigen but only one of them was determined in some of our patients, we considered both antibodies together and called anti-TG2/EMA positive individuals to those showing either anti-TG2 or EMA positive antibodies. Among the 178 patients with available anti-TG2 level, 97% were considered positive, the vast majority showing elevated levels (69%). A severe villous lesion (Marsh 3b or Marsh 3c) was observed in 90% of the patients, being the majority Marsh 3c.

In a first exploration of the demographic, clinical and analytical variables, early onset was associated with classical clinical presentation: mean age 2.08 ± 0.09 years (range 7 months-14 years) in patients with classical symptoms vs. 6.21 ± 0.43 years (range 13 months-14 years) in patients with non-classical presentation (*p* < 0.001); and also with more severe tissue injury: 2.33 ± 0.51 in Marsh 2 (10 subjects), 3.98 ± 0.70 in Marsh 3a (29 subjects), 3.01 ± 0.22 in Marsh 3b (134 subjects) and 2.36 ± 0.13 in Marsh 3c (283 subjects) (*p* = 0.007); and with presence of anti-TG2/EMA antibodies: 1.31 ± 0.12 vs. 3.27 ± 0.18 in subjects with negative and positive antibodies, respectively (*p* < 0.001). Severity of mucosal lesion was also associated with sex, with males showing milder (Marsh 0 to Marsh 3a) mucosal lesions (*p* = 0.014) independently of age at onset; and with anti-TG2 level (*p* = 0.013), independently of age at onset and sex. To note, only two patients showed Marsh 0 and Marsh 1, both EMA-positive and with clinical and analytical response to the gluten free diet. After gluten challenge, the patient with Marsh 0 showed positive anti-TG2 antibodies and atrophy (Marsh 3b).

Next, we considered the association between clinical and analytical features with the HLA-DQ status.

A family history of CD was observed in 22 (4.8%) of our patients. Their HLA-DQ distribution is shown in Table 2.

**Table 2:** HLA-DQ distribution in our patients with a family history of celiac disease

HLA genotype	N (%)
HLA-DQ2.5/DQ2.5	6 (27.3)
HLA-DQ2.5/DQ2.2	8 (36.4)
HLA-DQ2.5 /DQX	7 (31.8)
HLA-DQ8/DQ8	1 (4.5)

Comparison between these HLA-DQ frequencies and the ones observed in the remaining patients showed a nearly significant result: *p* = 0.067. Specifically, HLA-DQ2.5 and HLA-DQ8 homozygosity were notoriously increased in the group of patients with CD relatives: 27.3% vs. 10.9% (*p* = 0.03) and 4.5% vs. 0.2% (*p* = 0.093), respectively, although a significant result was only achieved when considering HLA-DQ2.5. These results led us to perform subsequent analyses after stratifying by family history of CD.

No significant changes across HLA-DQ genotypes were observed between sexes. Next, we analyzed the influence of HLA-DQ on age at onset. In the group with non-CD family history, we observed a significantly later disease onset in HLA-DQ8 carriers (including HLA-DQ8/DQ8 and HLA-DQ8/DQ2.5 subjects) when compared to non-HLA-DQ8 carriers: mean age 4.43 ± 0.66 years vs. 2.54 ± 0.11 years, respectively (*p* < 0.001). In patients with family history of CD, we could not evaluate this issue since HLA-DQ8 was only observed in one patient.

Finally, we studied the possible influence of the double HLA-DQB1*02 gene dosage (in HLA-DQ2.5 carriers) on clinical presentation, serology and histological severity (Table 3).

**Table 3:** HLA-DQ data in relation to clinical and analytical features stratified by family history in CD

		Sex *n* = 463		Clinical Presentation n = 463		Histological Damage *n* = 458		Anti-TG2/EMA *n* = 275		Anti-TG2 Levels *n* = 172	
		M	F	Classical	Non-Classical	M3b + M3c	Milder lesion	Positive	Negative	≥100	< 100
Family History	HLA-DQ2.5 double dose	10 66.7%	5 33.3%	4 28.6%	10 71.4%	13 92.9%	1 7.1%	12 100%	0	6 75%	2 25%
	Other HLA-DQ	3 42.9%	4 57.1%	3 37.5%	5 62.5%	8 100%	0	5 100%	0	3 60%	2 40%
Non-Family History	HLA-DQ2.5 double dose	68 39.1%	106 60.9%	162 92.6%	13 7.4%	164 94.8%	9 5.2%	110 99.1%	1 0.9%	43 70.5%	18 29.5%
	Other HLA-DQ	94 35.2%	173 64.8%	229 86.1%	37 13.9%	232 88.2%	31 11.8%	153 93.3%	11 6.7%	67 68.4%	31 31.6%

Slight variations in the number of patients depending on the considered variables were present owing to missing data. In the non-CD family history group, the classical clinical presentation was more frequently observed in HLA-DQ2.5 patients with double HLA-DQB1*02 dosage, and the effect depended on age at onset (Table 4).

**Table 4:** Association between HLA-DQB1*02 gene dosage and clinical and analytical features

	Bivariate analysis		Multivariate analysis	
	OR (CI 95%)	p	OR (CI 95%)	p
Clinical presentation^a^	2.01 (1.04–3.91)	0.04	2.31 (1.01–5.32)*	0.05*
Histological severity^b^	2.43 (1.13–5.26)	0.02	2.54 (1.16–5.52)**	0.02**
Serology^c^	6.64 (0.84–52.7)	0.07	6.09 (0.74–49.87)**	0.09**
Anti-TG2 level^d^	0.91 (0.45–1.81)	0.78	0.91 (0.45–1.61)**	0.80**

*corrected by age at onset. When corrected by sex and age: OR (95% CI) = 2.2 (0.95–5.10). *p* = 0.066;

**corrected by age at onset and sex

ORs are referred to ^a^ classical vs. non-classical presentation; ^b^ Marsh 3b/Marsh 3c vs. others; ^c^ positive vs. negative anti-TG2/EMA, excluding IgA deficient patients; ^d^ anti-TG2 ≤ 10 vs. > 10 the upper limit of normality

This genetics was also associated with the severity of histological damage, being this effect not probably affected by age and/or sex. The presence of specific antibodies was increased in presence of two HLA-DQB1*02 alleles, although the effect was only nearly significant. Anti-TG2 levels were not associated with HLA gene dosage. The effect of the HLA on clinical presentation and histological severity was also analyzed adjusting by anti-TG2 titer in the subgroup of patients with available data, but this variable was not acting as a confusing variable (data not shown).

No significant differences were observed in the group of family history, but our results are compromised by the small sample size and the resulting low statistical power.

No differences between HLA-DQ2.5 heterozygous individuals (excluding HLA-DQ2.5/DQ8 subjects) and HLA-DQ8 carriers were observed regarding any of the considered features (age at onset, clinical presentation, antibodies or histological damage) after adjustment for age.

## Discussion

We have studied, to the best of our knowledge, the largest CD series in relation to HLA and clinical and analytical features. A total of 463 children were considered, with a predominance of girls, early onset and classical clinical presentation, as commonly reported in pediatric series with CD [14, 18–20]. Also according to the literature, we found that early onset was associated to classical symptoms. It is well-known that a classical presentation is the predominant clinical form in children, especially in those under 3 years old [21]. A higher frequency of the non-classical clinical presentation was observed in patients with a family history of CD. This observation must be cautiously interpreted, because in absence of clinical symptoms suggestive of CD, the screening for this disease is performed in individuals belonging to risk groups, which include individuals having first-degree relatives with CD. However, it has been previously observed that CD patients with affected first-degree relatives developed CD after years of negative serological testing, being all HLA-DQ2.5 positive and clinically silent for CD [22].

We found a lower percentage of positive serology in children under 2–3 years old, as well as a positive association of anti-TG2 levels and mucosal severity. Both observations are already well-known [12, 23] and the last one has indeed been used to avoid biopsy in those children with high antibody titers according to the most recent ESPGHAN diagnostic guidelines [1]. In addition, we found more severe histological damage at earlier onset, as also previously reported [24], and in females.

When considering the influence of HLA-DQ on CD features, the first notorious observation was the higher frequency of HLA-DQ2.5 and HLA-DQ8 homozygotes in individuals with first-degree relatives with CD. This could be an expected result considering the high heritability of CD and that HLA accounts for around 40% of the total genetic risk.

The observed different genetic background depending on the family history of CD forced the subsequent analyses based on the role of HLA on clinical and analytical features, to be performed in the two groups separately. The most numerous group lacked CD familiarity. In this case, we observed that a later disease onset was present in HLA-DQ8 carriers, including those who were HLA-DQ2.5/DQ8. Although, to our knowledge, this is the first report of this effect, it is noteworthy to mention that Liu et al. [11] studied prospectively CD onset in children with HLA-DQ2.5 or HLA-DQ8 and observed that HLA-DQ2.5 homozygous subjects showed the earliest CD onset, being the other groups studied: HLA-DQ2.5/DQ8, HLA-DQ8/DQ8 and HLA-DQ8/non-HLA risk. Striking observations also emerged when studying the differential effect of HLA-DQ2.5 with double dose. The two groups established to make these comparisons (HLA-DQ2.5 carriers with two HLA-DQB1*02 alleles vs. the remaining patients) constitute the largest ones and give the most statistical power. We found that a classical clinical presentation was increased in carriers of HLA-DQ2.5 with double HLA-DQB1*02 dose. A similar effect was described by Zubillaga et al. [14] and Piccinni et al. [13], the last ones showing that atypical or silent/latent CD was more frequent in patients with low risk genetics. We also observed an association of HLA-DQ2.5 with double dose with severity of mucosal lesion, concordantly with previous studies in children and adults [9, 12]. The association with the presence of anti-TG2/EMA antibodies was only nearly significant, nevertheless, similar results were previously described in adults [25]. We did not find association of HLA-DQ with anti-TG2 levels, which has been also reported [11, 12, 26]. This could be due to the way of analyzing data, since we considered only two anti-TG2 levels: < or ≥ 10 times the upper limit of normality, because that was the only information available for most of our patients. Further analyses using the precise values of anti-TG2 antibodies could yield different results and need to be considered.

Only 22 individuals showed affected relatives and therefore the HLA influence could not be evaluated properly in this subgroup. However, it seems that HLA-DQ2.5 with double dose does not lead to a classical clinical presentation, which would differentiate both groups. Regarding the remaining considered features, it is possible that similar results exist. In a previous work analyzing 144 CD adults with CD family history, Karinen et al. observed a gene dosage effect of HLA-DQB1*02 on the grading of villous atrophy and age at onset [10].

CD is a highly heterogeneous disorder. When considering clinical manifestations, it ranges from a subclinical disease to a wide variety of signs and symptoms. Similarly, all the possible range of values is observed when looking at specific antibody levels. The intestinal damage, considered the hallmark of CD, can also vary from intraepithelial lymphocytosis to total atrophy. In this work, we demonstrate that HLA-DQ gene dosage is contributing to the observed heterogeneity. The highest genetic risk, which is conferred by the presence of the HLA-DQ2.5 heterodimer with two copies of HLA-DQB1*02 is associated to an earlier onset, a classical clinical presentation and a severe histological damage. It could also have influence on the presence of antibodies targeting TG2. The correlation between some of those parameters was previously known, and with our work, HLA becomes part of the picture. This issue has been largely suspected, but many apparent discrepancies are present among previous studies, mostly influenced by the small sample sizes previously considered, which may contribute to obtain false negative results [27]. Besides our sample size, other strength of our study is that all patients were diagnosed in the same center and revised by the same physician. Therefore no bias by different clinical practice exists.

Further studies are necessary to ascertain the impact of our observations in patients with a family history of CD and in adults, although looking at previous reports, it is possible that these associations are also present.

It may be that differences also exist between CD patients with HLA-DQ2.5 with single dose and those carrying HLA-DQ8 or lower risk HLA genotypes. The frequency of patients with these genetics is very low, and a multicenter study would be necessary to address this issue.

## Conclusions

The current use of HLA genotyping in clinical practice relies on its high negative predictive value. It is recommended to test HLA in patients with an uncertain diagnosis, as those with negative CD-specific antibodies and/or mild histological changes in small intestine. Based on the associations here found between double dose HLA-DQ2.5 and some clinical and analytical parameters, most probably high risk HLA-DQ genotypes will not be present in those subjects, which should be considered by physicians. Our study also suggests that patients with a family history of CD need a special consideration, since HLA-DQ status show slight variations.

HLA-DQ genotypic frequencies differ slightly between CD patients depending on their family history of CD. In patients lacking CD first-degree relatives, carriage of HLA-DQ2.5 with double dose of HLA-DQB1*02 seems to be associated with classical clinical presentation and more severe histological damage.

## Data Availability

The datasets used and analyzed during the current study are available from the corresponding author on reasonable request.

## Abbreviations

CD: Celiac disease
EMA: Anti-endomysium antibodies
PCR-SSOP: Polymerase Chain Reaction-Sequence Specific Oligonucleotide Probe
TG2: Transglutaminase 2
